# The regulatory network of potential transcription factors and MiRNAs of mitochondria-related genes for sarcopenia

**DOI:** 10.3389/fgene.2022.975886

**Published:** 2022-09-12

**Authors:** Wanrui Fu, Guzailinuer Kadeer, Yaqi He, Ying Feng

**Affiliations:** Department of Nutrition, Huadong Hospital Affiliated to Fudan University, Shanghai, China

**Keywords:** sarcopenia, mitochondria, transcription factor, miRNA, WGCNA

## Abstract

**Background:** Mitochondrial dysfunction is a significant contributor to sarcopenia, but the mechanism remains unclear.

**Methods:** In the present study, we downloaded GSE117525 and GSE8479 datasets from Gene Expression Omnibus (GEO), then the weighted correlation network analysis (WGCNA) was used to construct scale-free co-expression networks respectively. The key genes of aging muscle were obtained by overlapping key modules of two networks. Receiver operating characteristic (ROC) curve was drawn to explore the diagnostic efficacy of key genes. Finally, a transcription factor-key gene network was constructed based on ChEA3 platform and hTFtarget database, and a miRNA-key gene network was constructed using starBase and the multimiR R package.

**Results:** The most positively or negatively correlated modules of the two datasets were identified, and genes related to oxidative phosphorylation and mitochondrial ribosomal proteins were identified as key genes. The diagnostic values were confirmed with ROC curves by self-verification (GSE117525 and GSE8479) and external verification (GSE47881). Then, Yin Yang 1 (YY1) was identified as the most important transcription factor of the transcription factor-key gene network. In addition, miRNAs related to key genes were also predicted.

**Conclusion:** The findings of the present study provide a novel insight into the pathological mechanism of sarcopenia.

## Introduction

Sarcopenia is defined as a progressive loss of skeletal muscle mass, strength, and function with advancing age (Papadopoulou, 2020). It occurs in approximately 5%–13% of older adults aged 60–70 years, and 11%–50% among those aged 80 or above (von Haehling et al., 2010). Meanwhile, the risk of weakness, falls, frailty, and fractures are increased in elderly individuals suffering from sarcopenia (He et al., 2021). Unfortunately, the etiology and pathogenesis of sarcopenia are still not fully elucidated, which reduces the efficacy and effectiveness of pharmacological interventions (Morley, 2016), and elucidation of the underlying pathological mechanisms may provide guidelines for sarcopenia prevention and therapy.

So far, a number of pathogenetic factors, such as mitochondrial dysfunction (Yang et al., 2021), inflammation (Schaap et al., 2009), denervation (Xu et al., 2021), and insulin resistance (González-Hedström et al., 2021) serve important roles in the development of sarcopenia. Among these mechanisms, it is believed that mitochondrial dysfunction plays a central role in the signaling networks of sarcopenia (Karam et al., 2017). As the metabolic hub for energy production, mitochondria provide adenosine triphosphate (ATP) for skeletal muscle contraction through oxidative phosphorylation (OXPHOS). However, in the process of cellular senescence, there are numerous changes in mitochondria, such as decreased mitochondrial membrane potential, increased proton leakage, and reactive oxygen species production (Amorim et al., 2022), which have been proven to have an important role in inflammatory responses (Marchi et al., 2022), insulin resistance (Hayden, 2022), as well as aging (Miwa et al., 2022). Mitochondrial dysfunction could lead to the onset and progression of sarcopenia, a better understanding of the regulation factors controlling mitochondrial gene expression level and driving mitochondrial dysfunction in sarcopenia is therefore needed.

For maintaining mitochondrial structure and function, normal gene expression encoding for mitochondrial proteins are required, which is controlled by transcriptional regulators, especially transcription factors (TFs) (Taylor and Bishop, 2022) and microRNAs (miRNAs or miRs) (Giordani et al., 2021). TFs act at the transcriptional level, while miRNAs act post-transcriptionally. TFs are proteins that bind to the upstream regulatory elements of genes (enhancers and silencers) to increase or decrease gene transcription and protein synthesis. Several nuclear transcription factors, such as cAMP response element-binding protein (CREB) (Lee et al., 2005) and signal transducer and activator of transcription 3 (STAT3) (Macias et al., 2014), have been identified to participate in the regulation of mitochondrial transcription. Additionally, miRNAs are short single-stranded noncoding RNAs with ∼22 nucleotides, and they can regulate the gene expression at the posttranscriptional level. Research has found that miRNAs have a role to play in affecting mitochondrial dynamics and bioenergetics and regulating the mitochondrial homeostasis, which is important for fine-tuning the muscle atrophy program (Soares et al., 2014; Cannataro et al., 2021). Therefore, elucidating the relationships between TFs and miRNAs as well as related networks in sarcopenia may provide new ideas for the development of clinical drug target and the prevention of disease.

With the development of gene chip technology, the weighted gene co-expression network analysis (WGCNA) method, a strong clustering algorithm, is widely used to detect gene co-expression modules. In this study, we employed WGCNA and other bioinformatic analyses for young vs. healthy older individuals based on Gene Expression Omnibus (GEO) database. Our results revealed that mitochondrial function genes coding for nicotinamide adenine dinucleotide (NADH) dehydrogenase subunits played an important role in skeletal muscle aging, and regulatory networks of TFs and miRNAs could be potential therapeutic targets in sarcopenia.

## Materials and methods

### Data collection and data preprocessing

Microarray data of GSE117525 and GSE8479 datasets were downloaded from GEO database (https://www.ncbi.nlm.nih.gov/geo/), and GPL20880 [HuGene-1_1-st] Affymetrix Human Gene 1.1 ST Array [HuGene11st_Hs_ENTREZG_18.0.0] and GPL2700 Sentrix HumanRef-8 Expression BeadChip platform was applied to analysis (Melov et al., 2007; Hangelbroek et al., 2016). GSE117525 dataset provided microarray data from vastus lateralis muscle biopsies of 53 young and 73 healthy older adults, and the GSE8479 dataset contained samples from the same position of 25 healthy older and 26 younger adults. In addition, the raw data were subjected to background correction and quantile data normalization using the Limma package in Bioconductor (http://bioconductor.org/packages/release/bioc/html/limma.html). The median absolute deviation (MAD) top 80% genes were selected for co-expression network construction.

### Co-expression network construction

The scale-free co-expression network was constructed using the weighted gene co-expression network analysis (WGCNA) package in R (Langfelder and Horvath, 2008). First, the goodSamplesGenes method of R software package WGCNA was used to remove outlier genes and samples. Then, according to the SoftThreshold, the appropriate power was used to generate a scale-free topology overlap matrix (TOM), and the corresponding dissimilarity (1-TOM) was also calculated. Next, the linkage hierarchical clustering was established based on TOM with a minimum size of 100. The Dynamic Tree-Cut algorithm was used to classify similar genes into one gene module.

### Identification of key modules and hub genes

First, module eigengenes (MEs) were the major components of a particular gene module, and the correlation between MEs and age was analyzed by Pearson Correlation Coefficient to distinguish the co-expression modules that presented high associations with skeletal muscle aging. Second, as a persuasive indicator for selecting a significant module, module significance (MS) was calculated according to the average value of gene significance (GS). Eventually, the co-expression modules having the highest associations with age were regarded as the key modules. The jVenn tool (Bardou et al., 2014) was adopted to identify the intersecting genes of key modules in GSE117525 and GSE8479 to obtain a set of hub genes.

### Functional and pathway enrichment analysis

To further explore the biological significance of hub genes on the pathogenesis of skeletal muscle aging, we executed gene ontology (GO) functional enrichment analysis and Kyoto Encyclopedia of Genes and Genomes (KEGG) pathway analysis by clusterProfiler R package (http://www.bioconductor.org/packages/release/bioc/html/clusterProfiler.html). An adjusted *p* < 0.01 was considered to be significantly enriched.

### Protein-protein interaction network analysis

Protein-protein interaction (PPI) information was accessed using the Search Tool for the Retrieval of Interacting Genes Database (STRING) (Szklarczyk et al., 2021) (https://www.string-db.org/), and a medium confidence score (≥0.4) was set as cutoff criteria. The results were converted visually by Cytoscape (version 3.7.1) software. The CytoHubba (version 0.1) and Molecular Complex Deletion (MCODE version 1.6.1) plug-ins for Cytoscape were employed to distinguish the top-ranked genes/nodes and modules with a cutoff MCODE score of >5.

### Validation of key genes

Genes in the module sifted out by MCODE were regarded as key genes. Next, key genes self-verification (GSE117525 and GSE8479) and external verification (GSE47881) were evaluated by area under the curve (AUC) of the receiver operating characteristic (ROC) curve by the pROC R package. In the GSE47881 dataset, 44 baseline muscle samples were obtained from healthy adults aged 18 to 75.

### Transcription factor enrichment analysis

ChIP-X Enrichment Analysis 3 (ChEA3) platform (Keenan et al., 2019) (https://maayanlab.cloud/chea3/) and hTFtarget database (http://bioinfo.life.hust.edu.cn/hTFtarget#!/target) were used for TF prediction of key genes respectively, and the common TFs and their targets were identified by the jVenn tool.

### Construction of microRNAs-mRNA network

The online database starBase (Li et al., 2014) and the multimiR R package (Ru et al., 2014) were used to predict the miRNAs, and the miRNA-mRNA network was constructed according to the common results.

## Results

### Weighted gene co-expression network analysis

**FIGURE 1:**
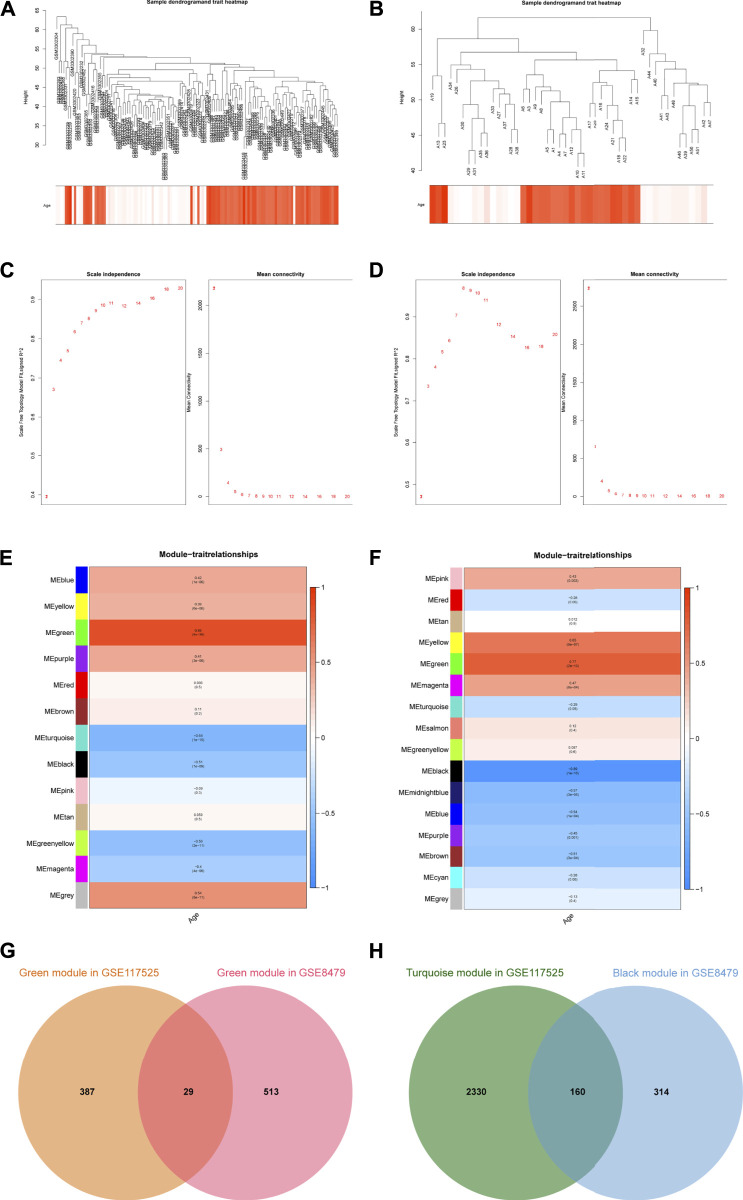
The key modules and hub genes identified by WGCNA. **(A,B)** Sample clustering after outliers were removed in GSE117525 **(A)** and GSE8479 **(B)**. **(C,D)** The soft-threshold power selecting processes. The *β* = 7 in GSE117525 **(C)** and *β* = 6 in GSE8479 **(D)** were selected when R^2^ reached 0.85. **(E,F)** Heatmaps of the correlation between module eigengenes and age in GSE117525 **(E)** and GSE8479 **(F)**. The corresponding correlations and *p*-values were presented. **(G)** The overlapped genes between the green modules. **(H)** The overlapped genes between the turquoise module and black module.

The WGCNA method was used to investigate the correlation between age and key genes. Before network construction, the outliers, GSM3302451 and GSM3302359 in GSE117525, as well as A49, A45, A2, and A20 in GSE8479, were removed (Supplementary Figure S1). The remaining samples were shown in Figures 1A,B. For the construction of the scale-free network, *β* = 7 and *β* = 6 were identified as the optimal soft-power value for GSE117525 and GSE8479 (Figures 1C,D). Then, we identified 13 modules of co-expressed genes in GSE117525 and 16 modules in GSE8479. Afterward, the correlations between modules and age were calculated. The green module had the strongest positive correlation with aging (*r* = 0.85), while the turquoise module had the strongest negative correlation (*r* = −0.64) in the GSE117525 database (Figure 1E). Meanwhile, the green module showed the strongest positive correlation (*r* = 0.77), and the black module had the strongest negative correlation (*r* = −0.89) in the GSE8479 database (Figure 1F). A set of hub genes were obtained by intersecting genes of key modules with the strongest relationship in GSE117525 and GSE8479 (Figures 1G,H).

### Identification of the shared pathways

**FIGURE 2:**
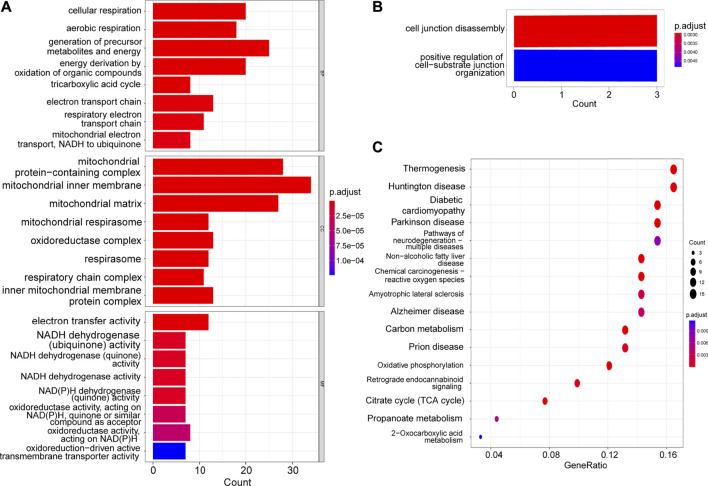
GO and KEGG enrichment of hub genes. **(A)** The GO analysis of the negatively-regulating genes. **(B)** The GO analysis of the positively-regulating genes. **(C)** The KEGG analysis of the negatively-regulating genes.

Using the GO and KEGG enrichment analyses, we further explored the common regulatory pathways that 29 positively-regulating and 160 negatively-regulating genes screened by WGCNA respectively. The GO analysis showed that the positively-regulating genes might be related to the cell junction disassembly and positive regulation of cell-substrate junction organization (Figure 2B). However, there was no KEGG pathway enriched in positively-regulating genes because the number of genes was too small. As for the negatively-regulating genes, the GO analysis showed that biological processes (BP) were enriched in cellular respiration, ATP metabolic process, mitochondrial electron transport, NADH to ubiquinone as well as fatty acid metabolic process, and cellular composition (CC) terms were enriched in mitochondrial matrix and NADH dehydrogenase complex, and molecular functions (MF) were enriched in electron transfer activity, and NADH dehydrogenase activity (Figure 2A). The KEGG analysis showed that these genes might be correlated with carbon metabolism, citrate cycle (TCA cycle), OXPHOS, and chemical carcinogenesis-reactive oxygen species (ROS) (Figure 2C).

### Construction and analysis of the protein-protein interaction network

**FIGURE 3:**
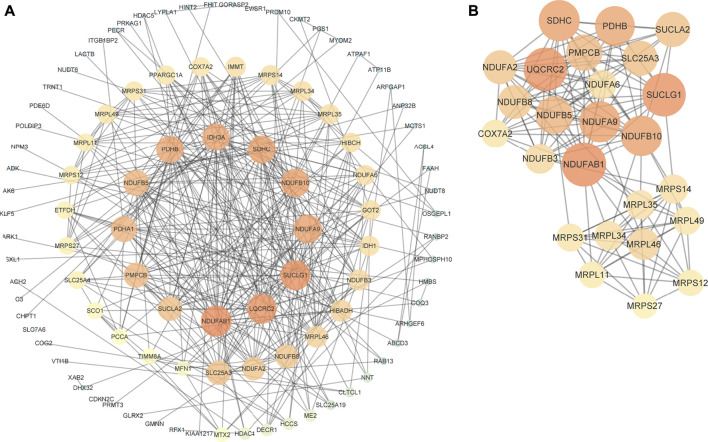
PPI network of negatively-regulating hub genes. **(A)** The core PPI network with a medium confidence score. **(B)** A significant module screened by MCODE. Different colors and sizes of nodes represent different degrees. The larger and brighter the node becomes, the greater the degree is.

To explore the interactions among negatively-regulating genes in depth, we constructed a PPI network using the STRING online database and Cytoscape software (Figure 3A). The core PPI network consisted of 110 nodes and 349 edges with a medium confidence score. Using Cytoscape MCODE, we found a significant module (score = 12.5) from the PPI network complex, and the degree algorithm in CytoHubba also showed genes in the module had top degrees (Figure 3B). Therefore, genes in this module (Supplementary Table S1) were regarded as key genes.

### Validation of key genes

Then, the diagnostic values of key genes were confirmed by ROC curve analysis and the AUC value. As shown in Table 1 and Supplementary Figures S2–S4, the AUC of 10 key genes was greater than 0.75 among three datasets, and the maximum AUC was 0.9646 for NADH:ubiquinone oxidoreductase subunit B8 (NDUFB8) in GSE8479. The results indicated that the key genes could play a good accuracy in the early diagnosis of sarcopenia.

**TABLE 1:** The AUC value of key genes in the three datasets.

Key gene	GSE117525	GSE8479	GSE47881
COX7A2	0.7454	0.8846	0.7586
MRPL11	0.6981	0.8115	0.8534
MRPL34	0.7844	0.9031	0.6638
MRPL35	0.7826	0.8615	0.694
MRPL46	0.6343	0.8369	0.8664
MRPL49	0.4885	0.9077	0.6142
MRPS12	0.8186	0.8000	0.7500
MRPS14	0.6648	0.8000	0.7349
MRPS27	0.6077	0.8354	0.6466
MRPS31	0.7612	0.7246	0.5841
NDUFA2	0.6190	0.8569	0.8168
NDUFA6	0.7707	0.8708	0.8168
NDUFA9	0.6198	0.8838	0.6918
NDUFAB1	0.7144	0.8592	0.7888
NDUFB10	0.6392	0.9262	0.7672
NDUFB3	0.6279	0.9092	0.5841
NDUFB5	0.8113	0.7931	0.7974
NDUFB8	0.6904	0.9646	0.6983
PDHB	0.7273	0.9092	0.5733
PMPCB	0.7565	0.7646	0.5841
SDHC	0.6663	0.9262	0.5754
SLC25A3	0.8842	0.6815	0.8384
SUCLA2	0.7842	0.9200	0.8125
SUCLG1	0.6795	0.9400	0.7845
UQCRC2	0.7697	0.9031	0.8125

### Construction of the transcription factor-key gene network

**FIGURE 4:**
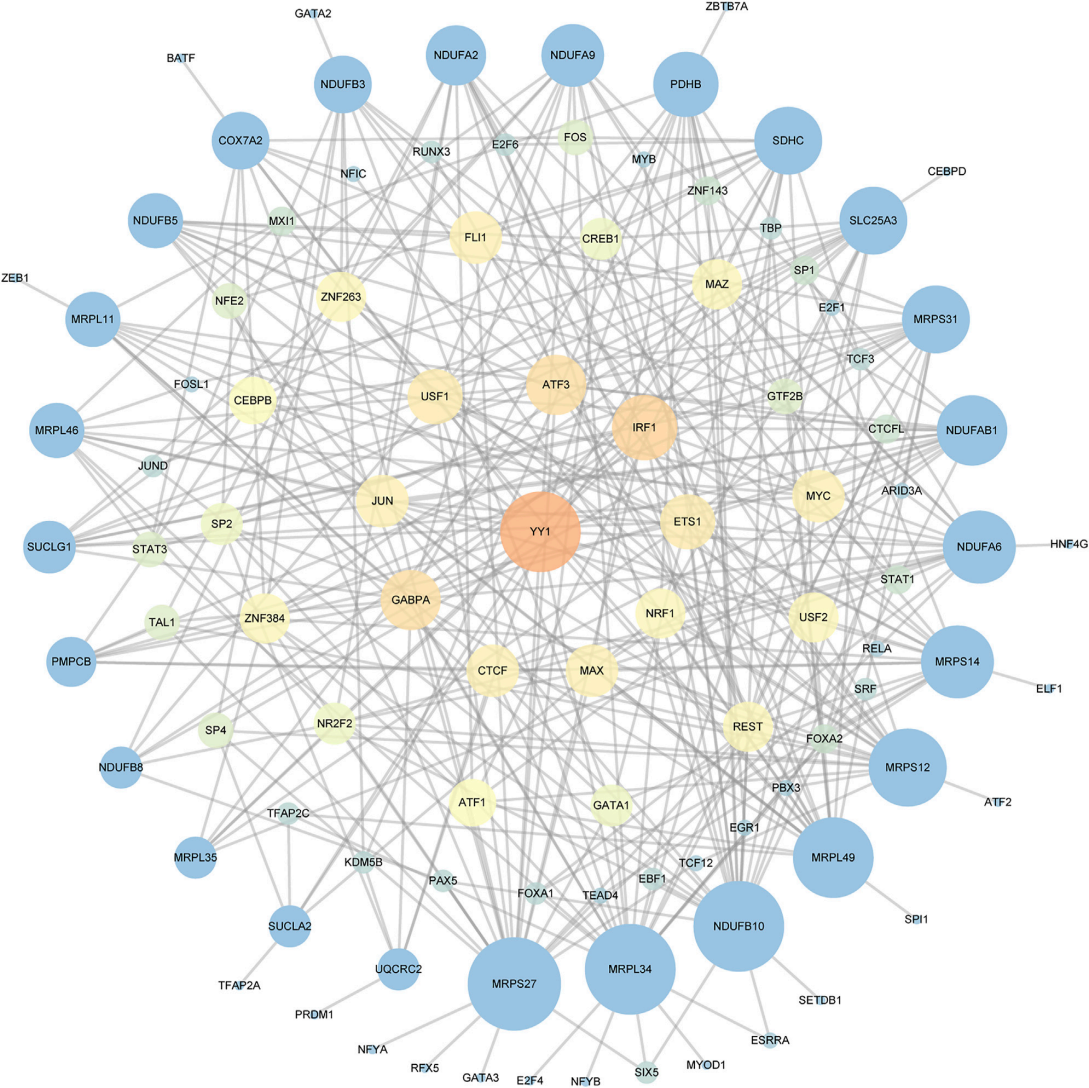
The TF-key Gene Network. Different colors and sizes of nodes represent different degrees. The larger and brighter the node becomes, the greater the degree is.

We used ChEA3 platform and hTFtarget database to predict the key transcription factor of key genes. As a result, there were 75 intersecting transcription factors enriched (Supplementary Table S2). As shown in Figure 4, twenty genes in all 25 key genes were predicted to be regulated by Yin Yang 1 (YY1).

### Construction of the microRNA-key gene network

**FIGURE 5:**
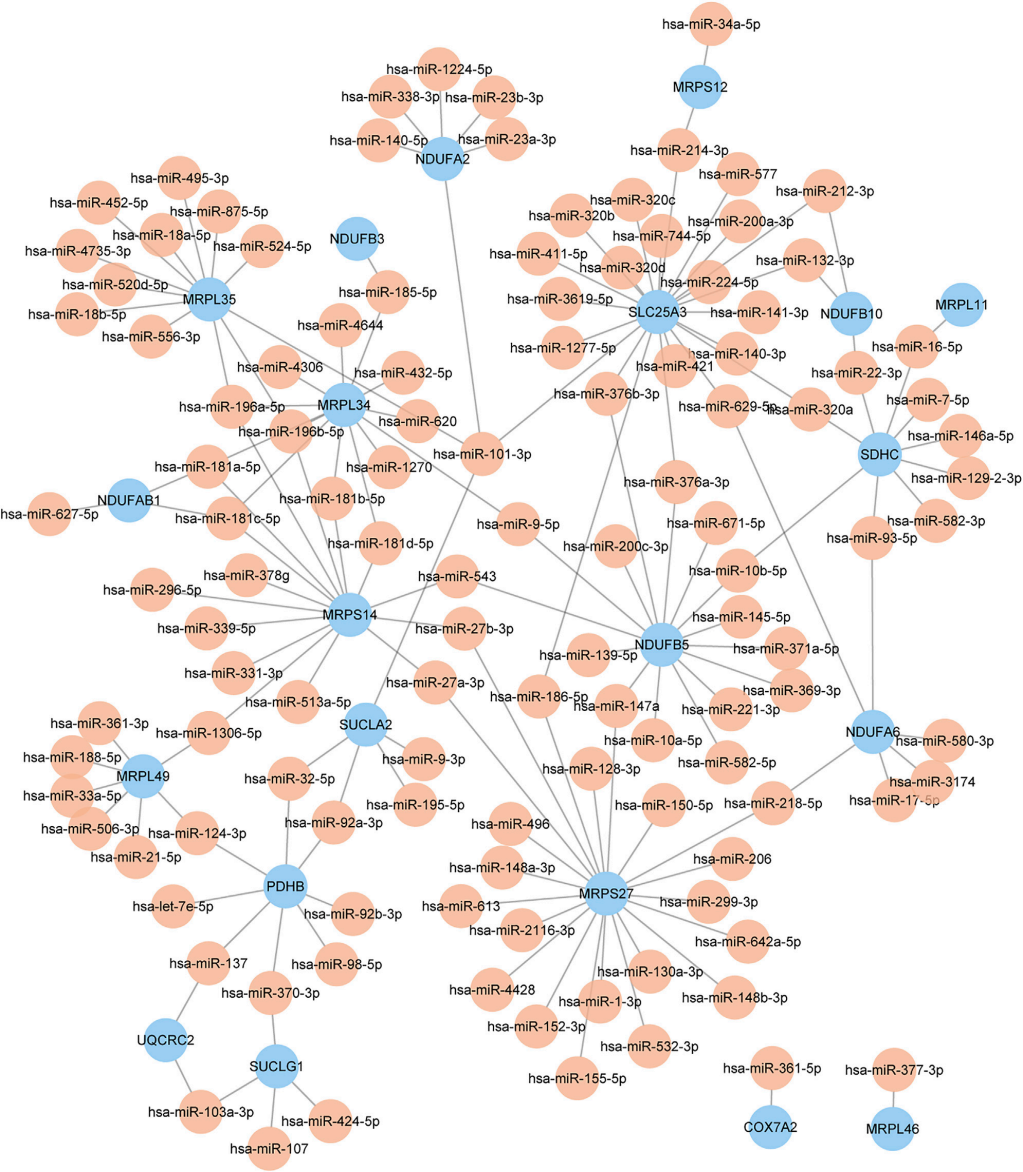
The miRNA-key gene network. Different colors of nodes represent different genes or miRNAs. The color yellow represents miRNA, and the color blue represents key genes.

**FIGURE 6:**
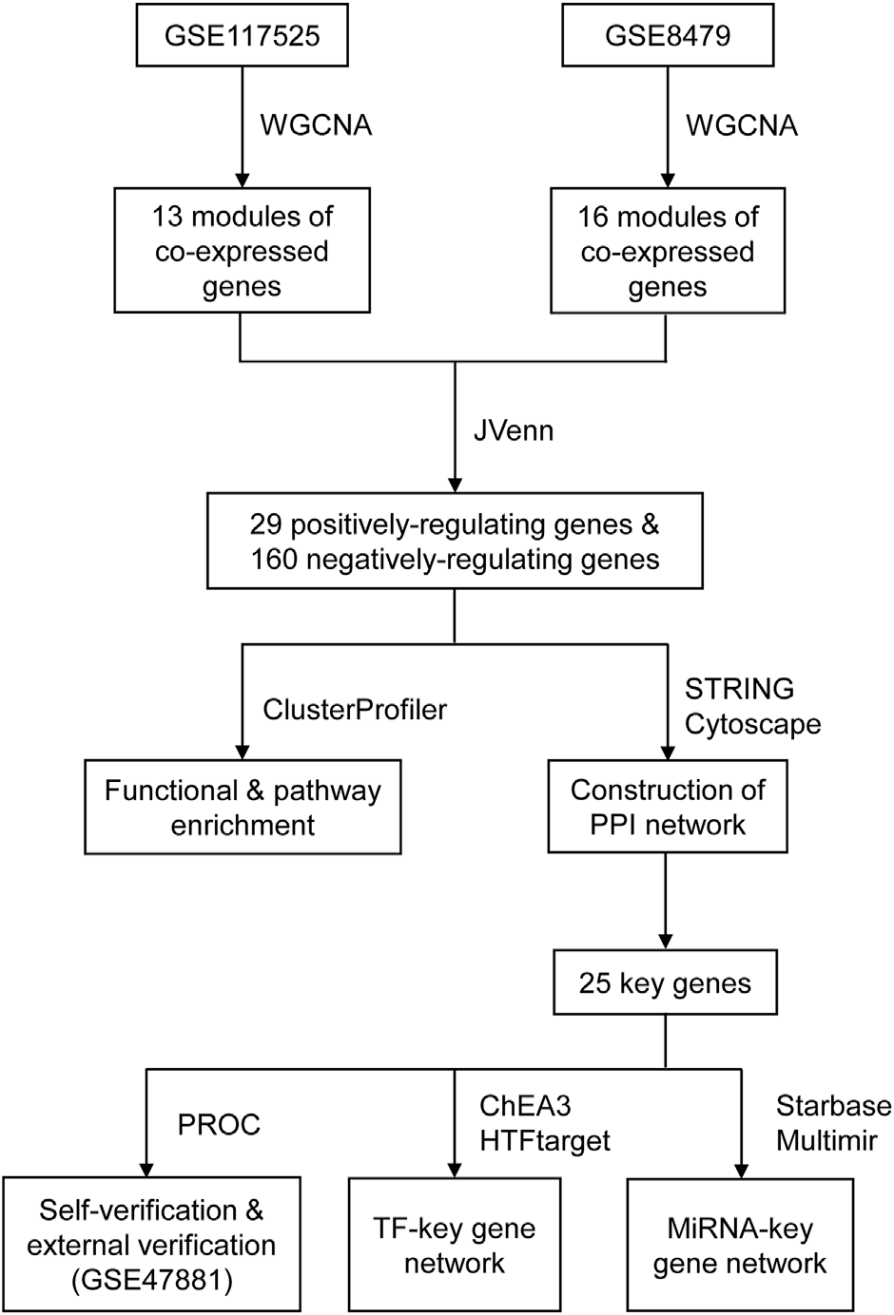
The flowchart of the analysis.

The miRNAs of key genes were identified using the online database starBase and the multimiR R package respectively, and 493 miRNAs were identified as candidates by starBase, as well as 583 miRNAs were identified by multimiR. To increase the accuracy, we selected the intersection of the results of each method (Supplementary Table S3). It should be noted that there was no corresponding miRNA for mitochondrial ribosomal protein S31 (MRPS31), NADH:ubiquinone oxidoreductase subunit A9 (NDUFA9), NDUFB8, and peptidase, mitochondrial processing subunit beta (PMPCB) after intersection. In the end, one hundred and thirty-nine miRNAs in total were obtained and the miRNA-key Gene Network was constructed as shown in Figure 5. In addition, the flowchart shows all essential and significant procedures in our study (Figure 6), and the abbreviations are provided in the supplementary abbreviation list (Supplementary Table S4).

## Discussion

Sarcopenia is very common in older adults. It is characterized by a progressive and generalized degenerative loss of skeletal muscle mass, quality, and strength with normal aging (Wiedmer et al., 2021). The pathogenesis of sarcopenia is complex, and it appears to be difficult the identification of novel specific molecular markers that can be accurately and effectively used in the diagnosis, treatment, or prognosis evaluation of sarcopenia. With the development of omics techniques and bioinformatics technology, new opportunities are offered to identify new targets to help us understand the pathophysiology of sarcopenia.

In this study, we constructed WGCNA to identify co-expression genes and possible biological pathways of sarcopenia based on the GSE117525 and GSE8479 datasets. The most positively and negatively correlated modules with aging were green and turquoise module in GSE117525 and green and black modules in GSE8479. The function analysis revealed that cellular respiration-related biological processes and pathways, including NADH to ubiquinone, thermogenesis, TCA cycle, and OXPHOS, were significantly involved in the overlapped genes between the most negatively correlated modules of the two datasets. Then, genes related to OXPHOS and mitochondrial ribosomal proteins were identified as key genes. YY1 along with the other 75 transcription factors was likely to play an important role in regulating the expression of key genes. In addition, as important regulators in the post-transcriptional regulation of gene expression, miRNAs in connection with key genes were predicted and the relevant miRNA-key gene network may help to explore the pathological mechanism of sarcopenia.

Our GO/KEGG results demonstrated that mitochondrial dysfunction played a significant role in the pathological process of sarcopenia. With advancing age, the mitochondrial capacity to produce energy decreases in skeletal muscle, particularly when maximal performance is required (Gonzalez-Freire et al., 2018). NAD decline has been recognized as one possible mechanism. As a coenzyme for redox reactions, NAD is at the center of energy metabolism, because NAD along with NADH participates in the TCA cycle and the electron-transporter channel, and an optimal NAD/NADH ratio is needed for efficient mitochondrial metabolism (Navas and Carnero, 2021). The decline in NAD levels is linked to numerous aging-associated diseases, including cognitive decline, metabolic disease, sarcopenia, and frailty (Covarrubias et al., 2021). Research has found that mitochondrial oxidative capacity and NAD biosynthesis are reduced in human sarcopenia across ethnicities (Migliavacca et al., 2019), and reducing NAD levels by ablating nicotinamide phosphoribosyltransferase (NAMPT)-mediated NAD salvage could lead to progressive muscle degeneration in the adult mouse (Frederick et al., 2016). Moreover, raising NAD (+) levels in old mice restores mitochondrial function to that of a young mouse in a sirtuin-1 (SIRT1)-dependent manner (Gomes et al., 2013). Therefore, targeting NAD metabolism has emerged as a potential therapeutic approach to ameliorate sarcopenia, and extend the human health-span and lifespan.

As the first and largest complex of the mitochondrial respiratory chain, NADH-ubiquinone oxidoreductase, or complex I, plays an important role in mitochondrial ATP synthesis. It could drive proton translocation across the inner mitochondrial membrane by liberating and transferring electrons from NADH to ubiquinone. Interestingly, some members of the NADH-ubiquinone oxidoreductase (NDUF) family were identified as key genes in our study, including NADH: ubiquinone oxidoreductase subunit A2 (NDUFA2), NADH: ubiquinone oxidoreductase subunit A6 (NDUFA6), NDUFA9, NADH: ubiquinone oxidoreductase subunit AB1 (NDUFAB1), NADH: ubiquinone oxidoreductase subunit B10 (NDUFB10), NADH: ubiquinone oxidoreductase subunit B3 (NDUFB3), NADH: ubiquinone oxidoreductase subunit B5 (NDUFB5), and NDUFB8, making this gene family into an important candidate for future study and biomarker development. Mutations in these genes could lead to complex I deficiency, which is one of the most frequent defects of the OXPHOS system. Complex I deficiency could result in the impairment of organs with a high-energy demand and participate in the pathogenesis of cancer, aging, and neurodegenerative conditions (Fiedorczuk and Sazanov, 2018). A study found that NDUFAB1 is a crucial regulator of mitochondrial energy and ROS metabolism as it could effectively enhance mitochondrial bioenergetics while limiting ROS production (Hou et al., 2019). In addition, the aging-associated reduction in expression of NDUFA6, NDUFA9, NDUFB5, NDUFB8, and NADH: ubiquinone oxidoreductase core subunit S2 (NDUFS2) was correlated with the decline in activity of OXPHOS in the senescent heart (Emelyanova et al., 2018). In conclusion, our findings indicated that genes coding for complex I were essential for a normal OXPHOS process, and they could be novel mitochondrial targets to prevent sarcopenia.

Apart from the NDUF family, many mitochondrial ribosomal proteins (MRPs) were also identified as key genes in this study. Mitochondrial ribosomes (mitoribosomes) consist of a small 28S subunit encoded by 30 MRP genes, and a large 39S subunit encoded by 52 MRP genes in human. They are implicated in the synthesis of all 13 mitochondrial DNA (mtDNA) -encoded protein subunits of the human OXPHOS system. Dysfunction of these proteins not only impairs mitochondrial protein translation but also causes primary mitochondrial respiratory chain activity deficiencies and clinical diseases, such as neurodegenerative diseases (Lopez Sanchez et al., 2021), inflammatory disorders (Gopisetty and Thangarajan, 2016) as well as aging (Molenaars et al., 2020). A study has found that MRPL49 shows altered proteolytic processing by dopamine treatment in Parkinson’s disease (Lualdi et al., 2019). In addition, decreased expression of MRPL12 could reduce mitochondrial OXPHOS in proximal tubular epithelial cells (Gu et al., 2021). In a word, MRPs are indispensable for respiration, but their biological functions are not yet clear and further research is needed.

In the present study, we constructed a TF-mRNA network to understand the potential mechanisms of sarcopenia. As a result, YY1 was screened out as the most important TF. When YY1 was deleted in skeletal muscle satellite cells, the acute damage-induced muscle repair was blocked, resulting in muscular dystrophy (Chen et al., 2019). And in another study, the muscle-specific knockout of Yy1 could lead to defective mitochondrial morphology and oxidative function (Blättler et al., 2012). Mechanistically, loss of YY1 or its repressive acetylation at K392/K393 may affect energy metabolism as mitochondrial complex I genes are bound directly by YY1 (Perekatt et al., 2014; Addicks et al., 2022). Notably, a study has found that YY1 is significantly upregulated in senior groups (Zampieri et al., 2015), and YY1 has been regarded as both a transcriptional repressor and activator (Kawamura et al., 2021). In the proliferative stage, YY1 is likely to inhibit the function of mitochondria, but in the differentiation stage, it switches to firing up mitochondria and OXPHOS (Blättler et al., 2012). The function of YY1 remains unclear, but the two-way regulation of YY1 on mitochondria during different periods may be an important mechanism for regulating the development and aging of muscle. The other TFs, such as interferon regulatory factor 1 (IRF1) (Baron et al., 2011), activating transcription factor 3 (ATF3) (Lim et al., 2006), CREB1 (Franko et al., 2008), also has been proved to participate in regulating the expression of genes encoding mitochondrial proteins during myogenesis and mitochondrial homeostasis.

MiRNAs are gene regulators that post-transcriptionally regulate specific genes. A growing stream of research suggests that miRNAs have a big role in skeletal muscle physiology by targeting and preventing the translation of specific mRNAs. Research has found that miR-127 and miR-434-3p were generally downregulated in most aged mouse muscles (Lee and Kang, 2022). Additionally, miR-101 was associated with frailty syndrome (Carini et al., 2022) and Alzheimer’s disease (Barbato et al., 2020). A study has found that miR-101a/b could suppress the p38 mitogen activated protein kinase (MAPK), interferon-gamma (IFN-gamma), and Wnt pathways and enhance the CCAAT/enhancer binding protein (C/EBP) pathway, which led to the inhibition of myoblast differentiation (Liu et al., 2021). Moreover, miR-101-3p has a regulative effect on mtDNA transcription and the assembly of the succinate dehydrogenase complex subunit C (SDHC) subunit, which is important for complex II biogenesis (Ziemann et al., 2022). In conclusion, miRNAs play vital roles in the regulation of OXPHOS and mitochondrial metabolism, providing new insights into deciphering the key molecular pathways related to sarcopenia and age-associated disorders.

Finally, the present study had several limitations that should be acknowledged: 1) Our study is limited to the processing of previous data, requiring *in vivo* and *in vitro* experiments to verify the results. 2) Due to the small number of overlapping positively-regulating genes, we could not identify KEGG pathways and key genes of them.

## Conclusion

In this study, genes related to OXPHOS and MRPs were regarded as the ones that had the strongest association with skeletal muscle aging. The relevant TFs and miRNAs were also identified and they could participate in the pathogenesis and treatment of sarcopenia by regulating the expression of mitochondria-related genes. In conclusion, our results will be helpful for better understanding the role of mitochondria in the pathophysiology of sarcopenia. Further mechanistic studies and more evidence are needed to validate these results and prove the mechanism.

## Data Availability

The original contributions presented in the study are included in the article/Supplementary Material, further inquiries can be directed to the corresponding author.
