# Supplementary figures and images for: The regulatory network of potential transcription factors and MiRNAs of mitochondria-related genes for sarcopenia
Source: Front Genet. 2022 Sep 12;13:975886. doi: 10.3389/fgene.2022.975886 (PMC9510666; doi:10.3389/fgene.2022.975886)

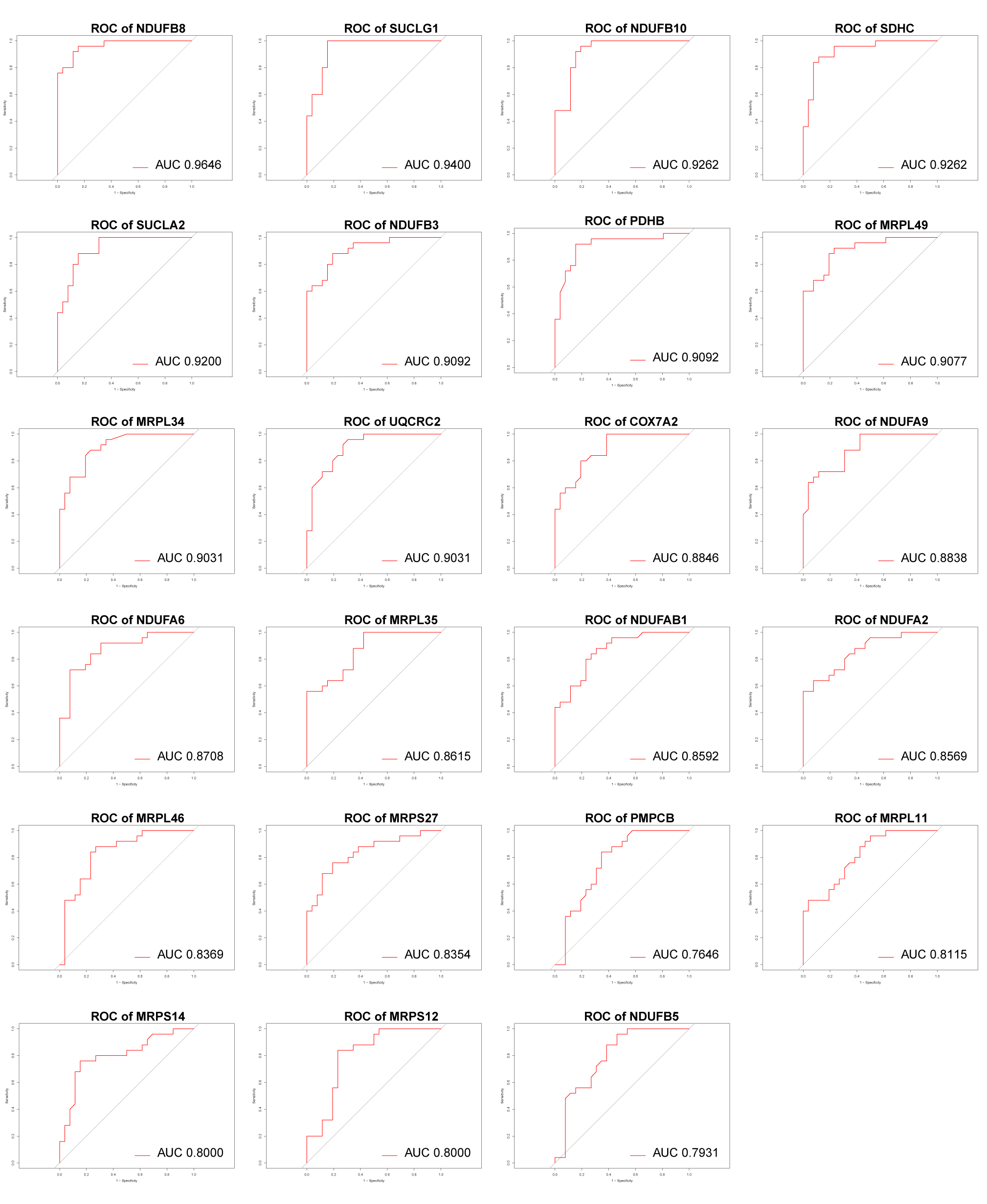

Supplement: Supplementary file 3 [file Image3.TIF]

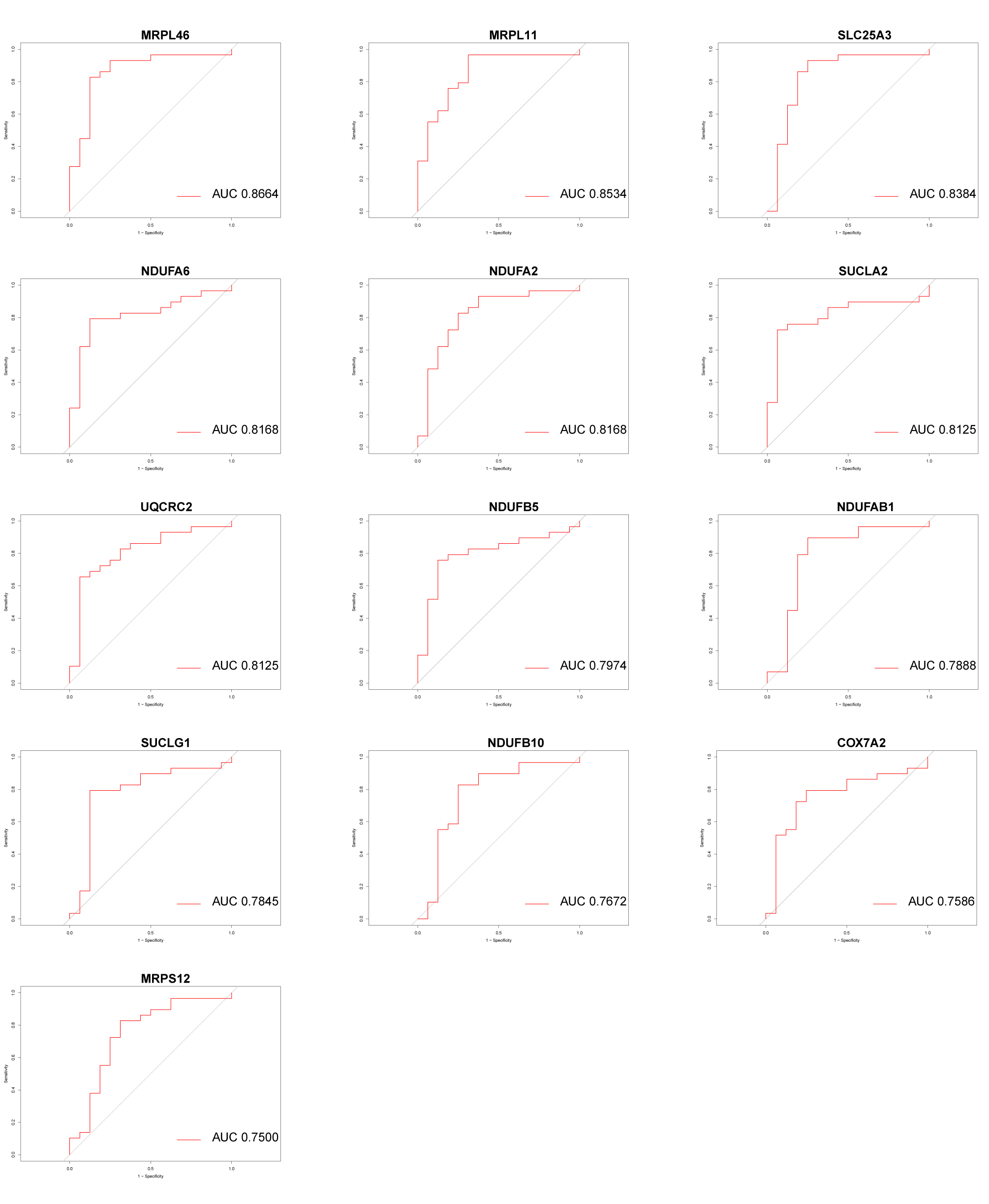

Supplement: Supplementary file 4 [file Image4.TIF]

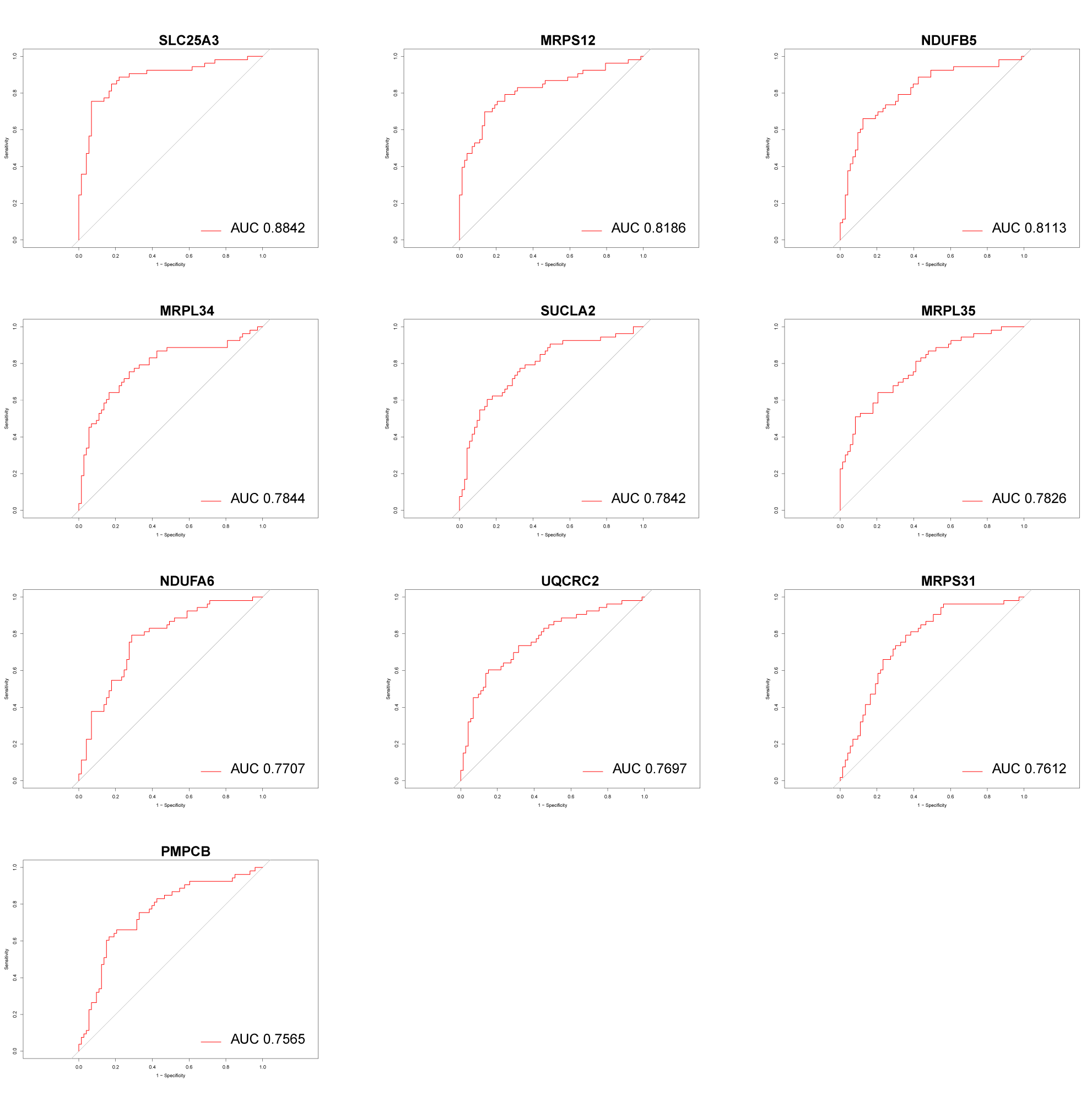

Supplement: Supplementary file 5 [file Image2.TIF]

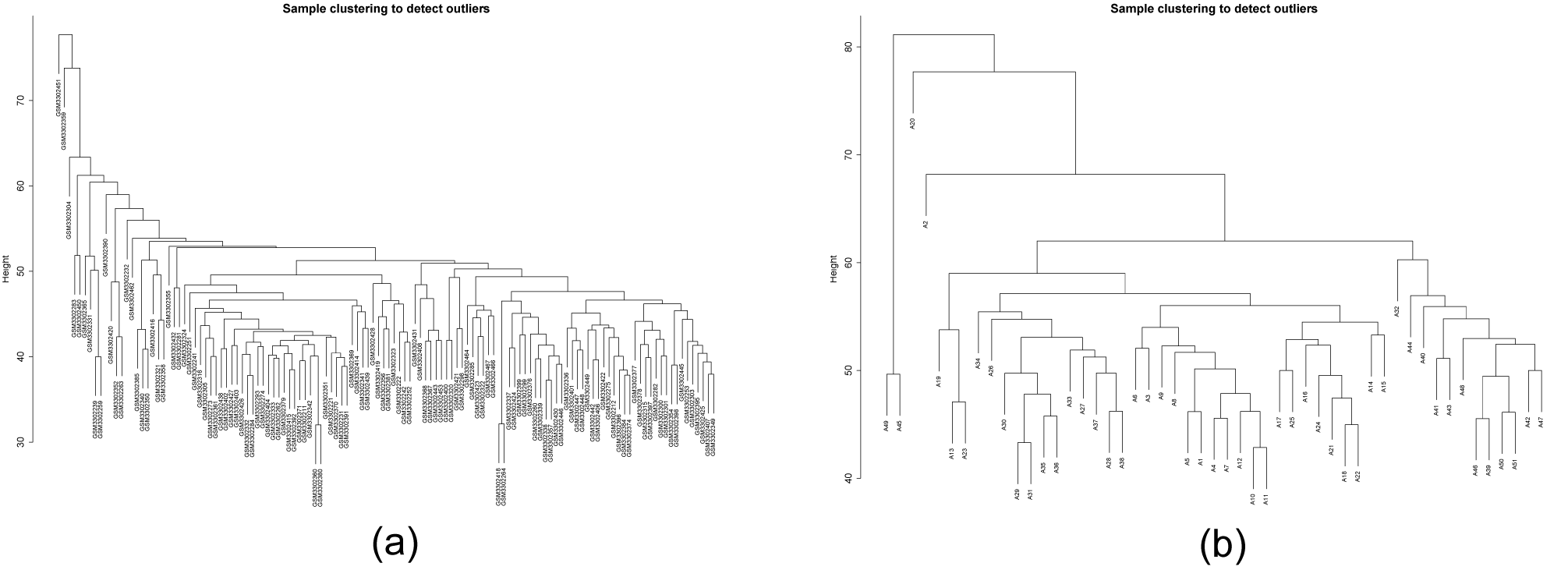

Supplement: Supplementary file 6 [file Image1.TIF]
