# Supplementary material for: The regulatory network of potential transcription factors and MiRNAs of mitochondria-related genes for sarcopenia
Source: Front Genet. 2022 Sep 12;13:975886. doi: 10.3389/fgene.2022.975886 (PMC9510666; doi:10.3389/fgene.2022.975886)
Supplement: Supplementary file 8 [file DataSheet1.docx]

The Regulatory Network of Potential Transcription Factors and MiRNAs of Mitochondria-related Genes for Sarcopenia

Supplementary File


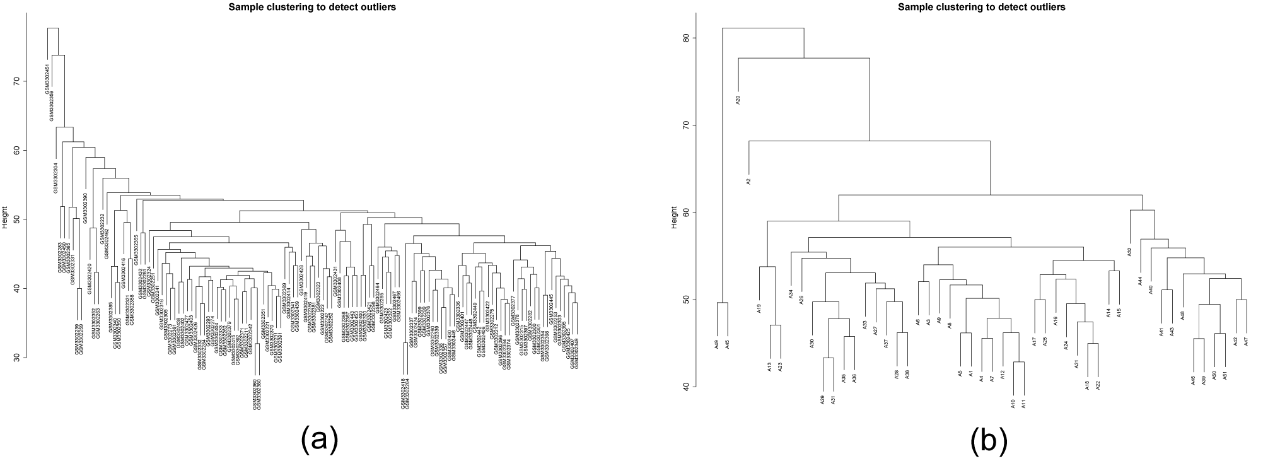


Figure S1. Sample clustering to detect outliers in GSE117525 (a) and GSE8479 (b).


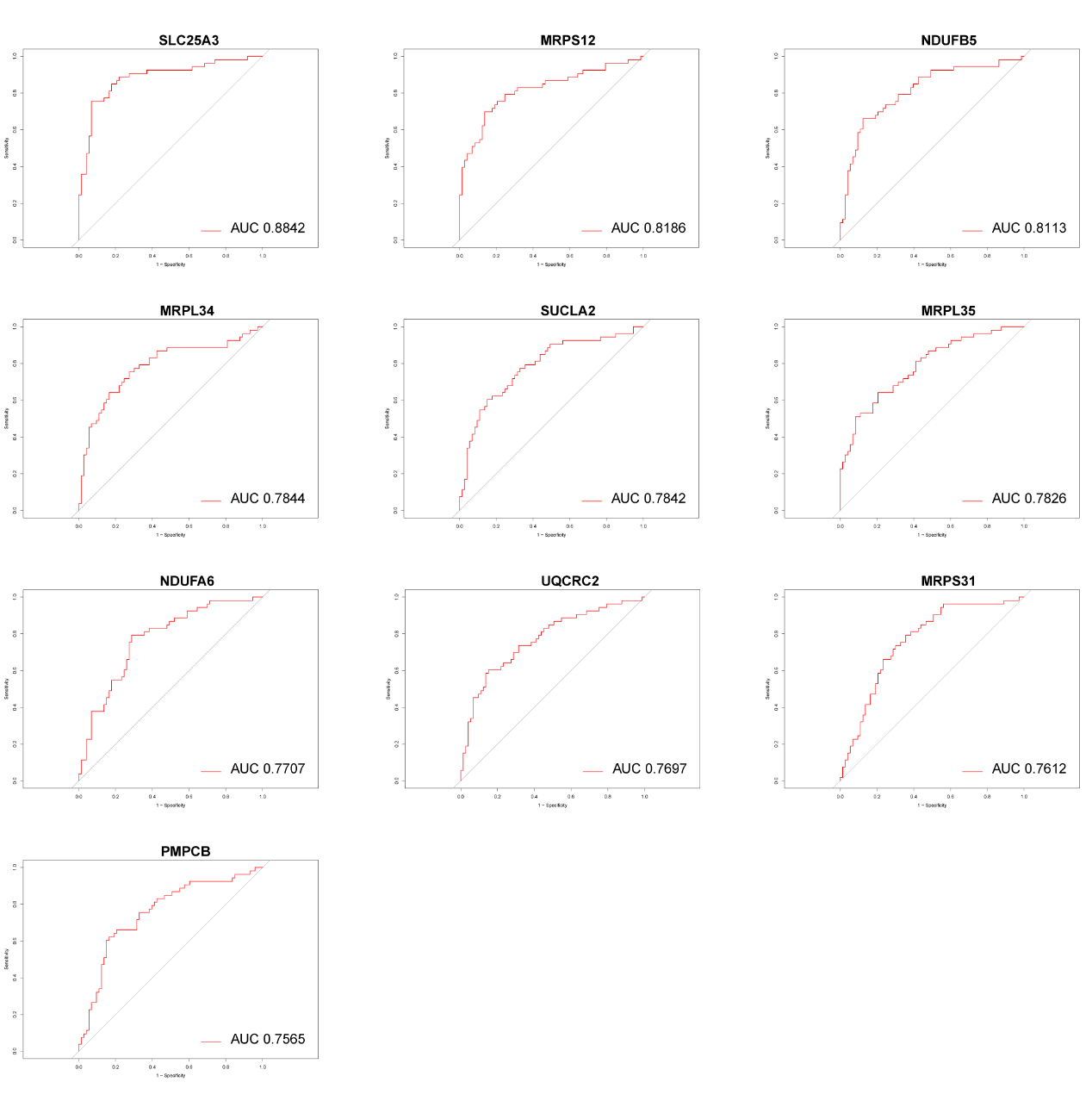


Figure S2. The ROC of the key genes in GSE117525. The figure contains only the key genes whose AUC ≥ 0.75.


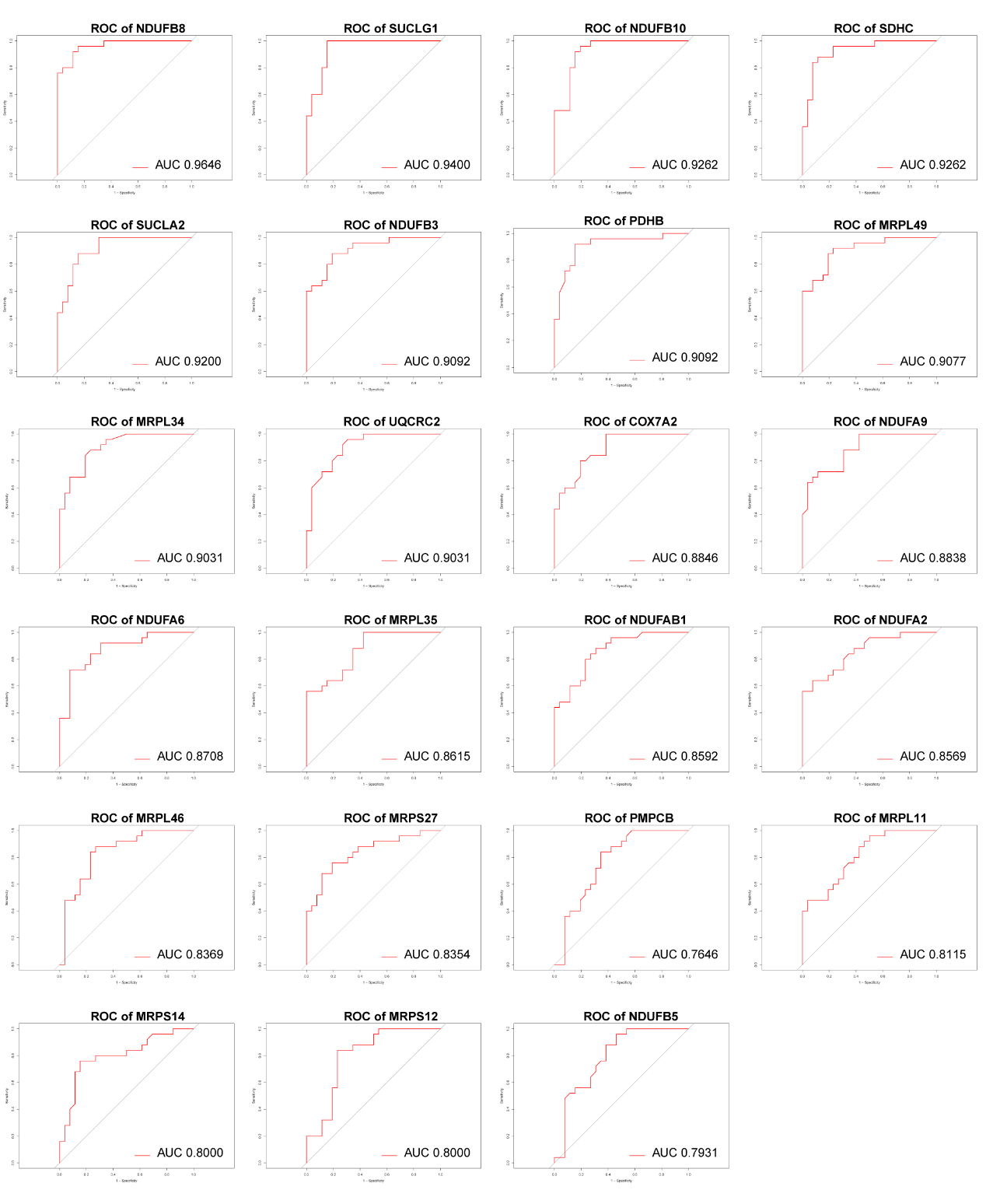


Figure S3. The ROC of the key genes in GSE8479. The figure contains only the key genes whose AUC ≥ 0.75.


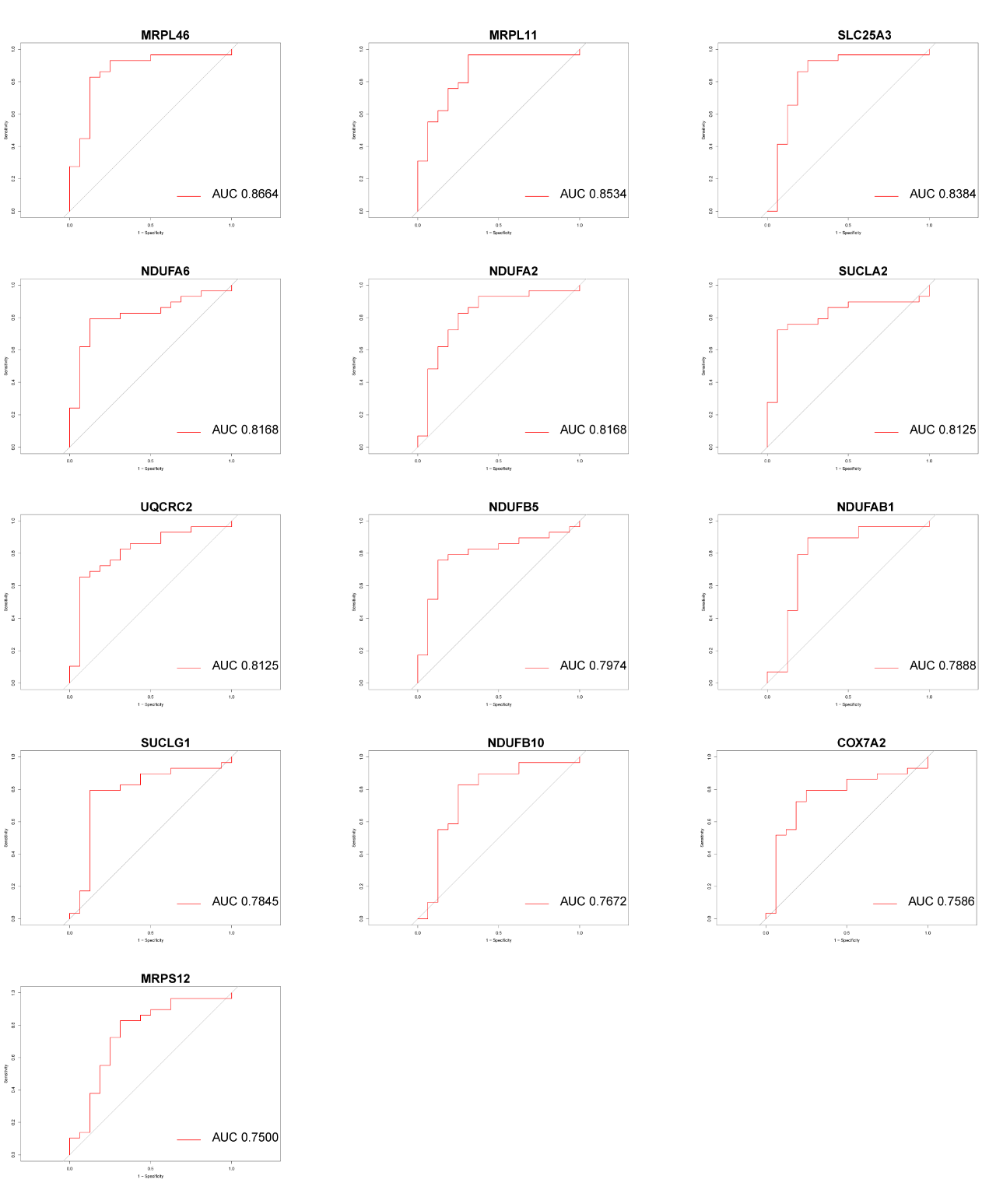


Figure S4. The ROC of the key genes in GSE47881. The figure contains only the key genes whose AUC ≥ 0.75.
